# Supplementary figures and images for: Tree seedling functional traits mediate plant-soil feedback survival responses across a gradient of light availability
Source: PLoS One. 2023 Nov 27;18(11):e0293906. doi: 10.1371/journal.pone.0293906 (PMC10681222; doi:10.1371/journal.pone.0293906)

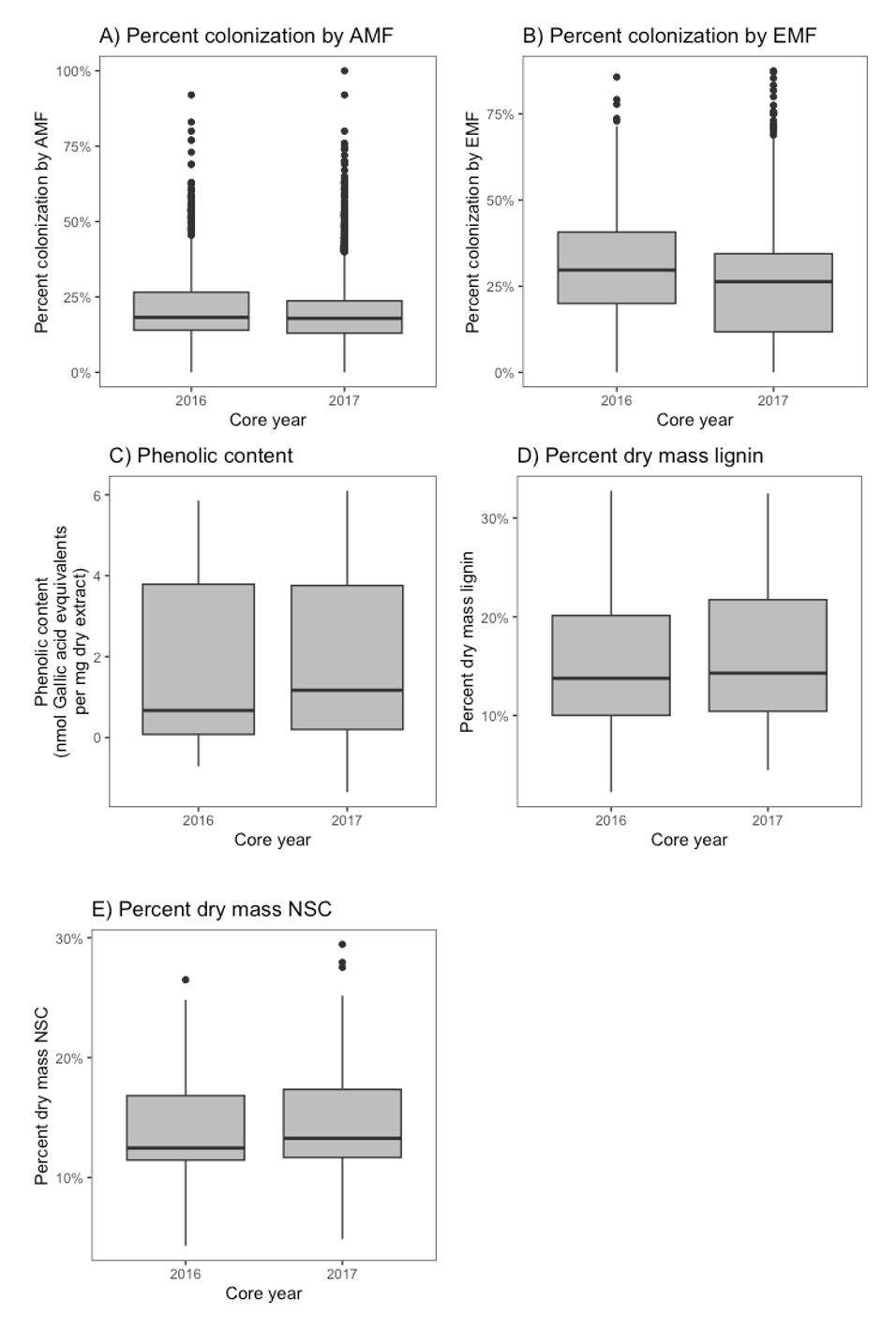

Supplement: S1 Fig — Prelimninary boxplots showing the effect of soil core year (2016, 2017) on tree seedling traits: A) AMF colonization, B) EMF colonization, C) Phenolics, D) Lignin, E) NSC. We were concerned that storing soil cores for an extended period of time would have potential negative effects on the microbial community. Specifically, we worried that the soil microbial community would be adversely affected. Preliminary analyses did not indicate any significant effect of soil collection year on seedling trait expression (AMF or EMF colonization, phenolics, lignin, NSC) or survival. (TIF) [file pone.0293906.s001.tif]

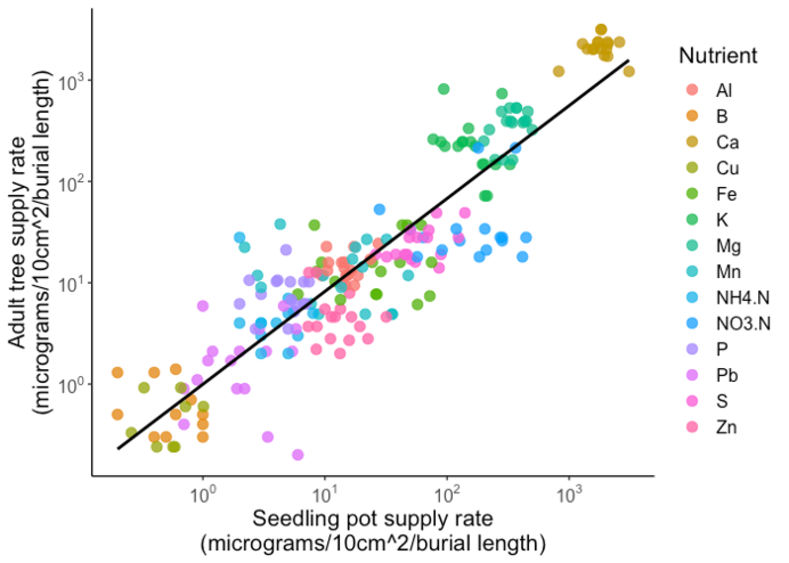

Supplement: S2 Fig — (TIF) [file pone.0293906.s002.tif]

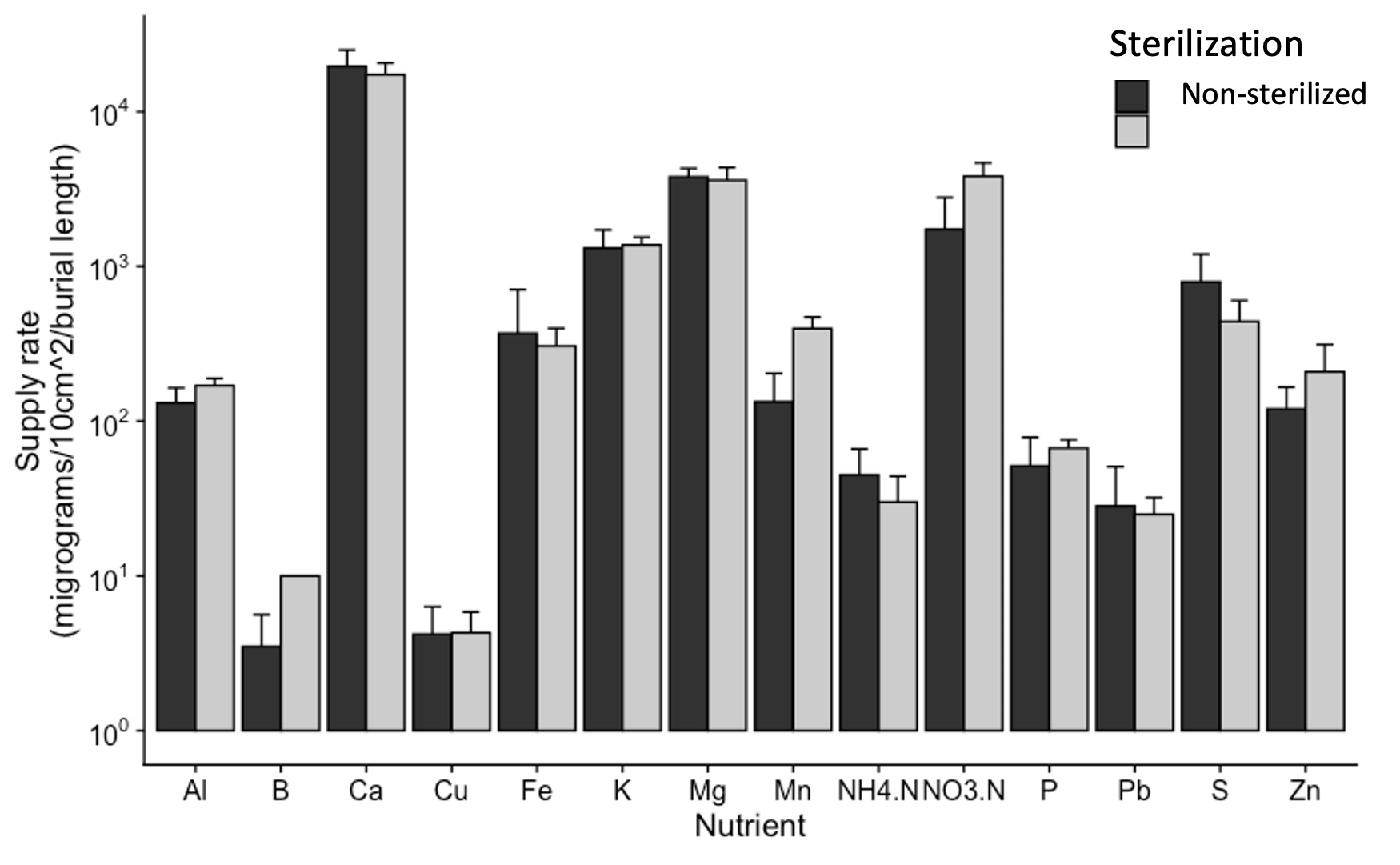

Supplement: S3 Fig — (TIF) [file pone.0293906.s003.tif]

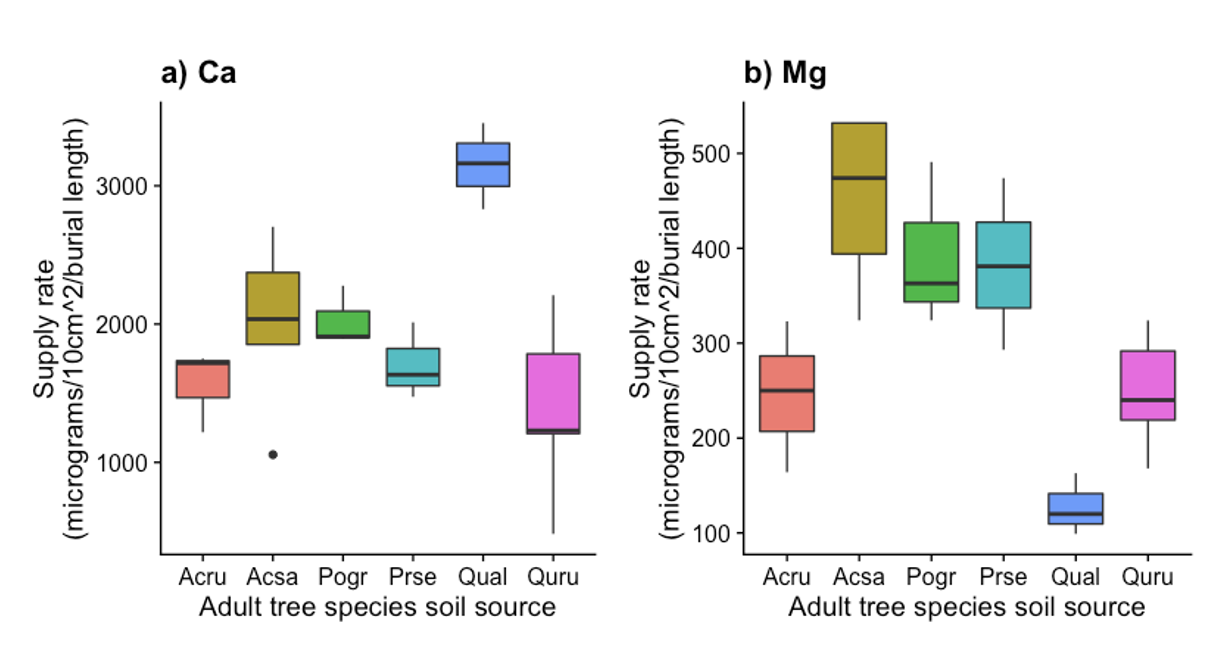

Supplement: S4 Fig — For a) Ca2+ and b) Mg2+. (TIF) [file pone.0293906.s004.tif]

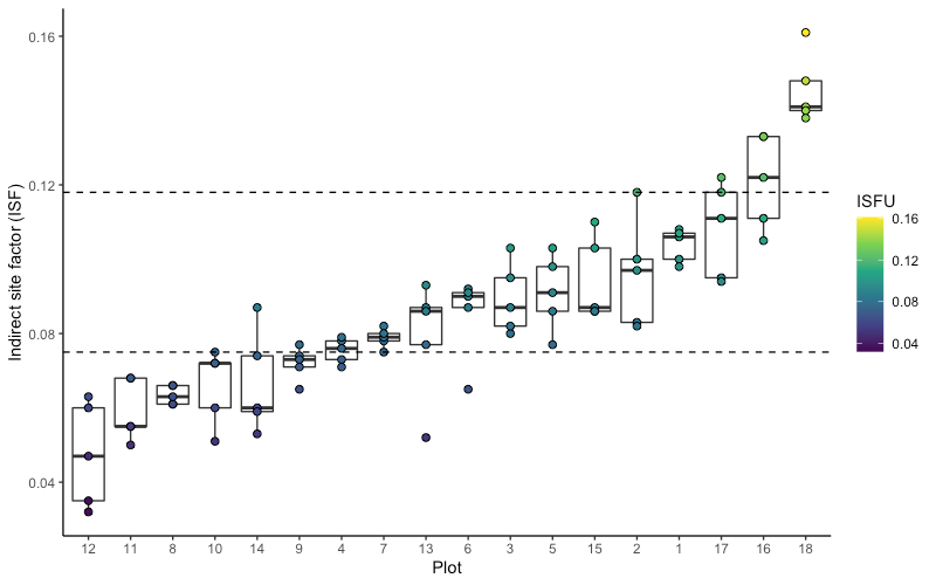

Supplement: S5 Fig — Indirect site factor (ISF, the proportion of diffuse solar radiation at a given location, relative to the amount of diffuse solar radiation in the open) in each subplot (n = 5) per common garden plot (n = 18). For analyses in which light availability was included as a categorical variable, low = 0.032–0.075 ISF, medium = 0.075–0.118 ISF, and high = 0.118–0.161 ISF. (TIF) [file pone.0293906.s005.tif]

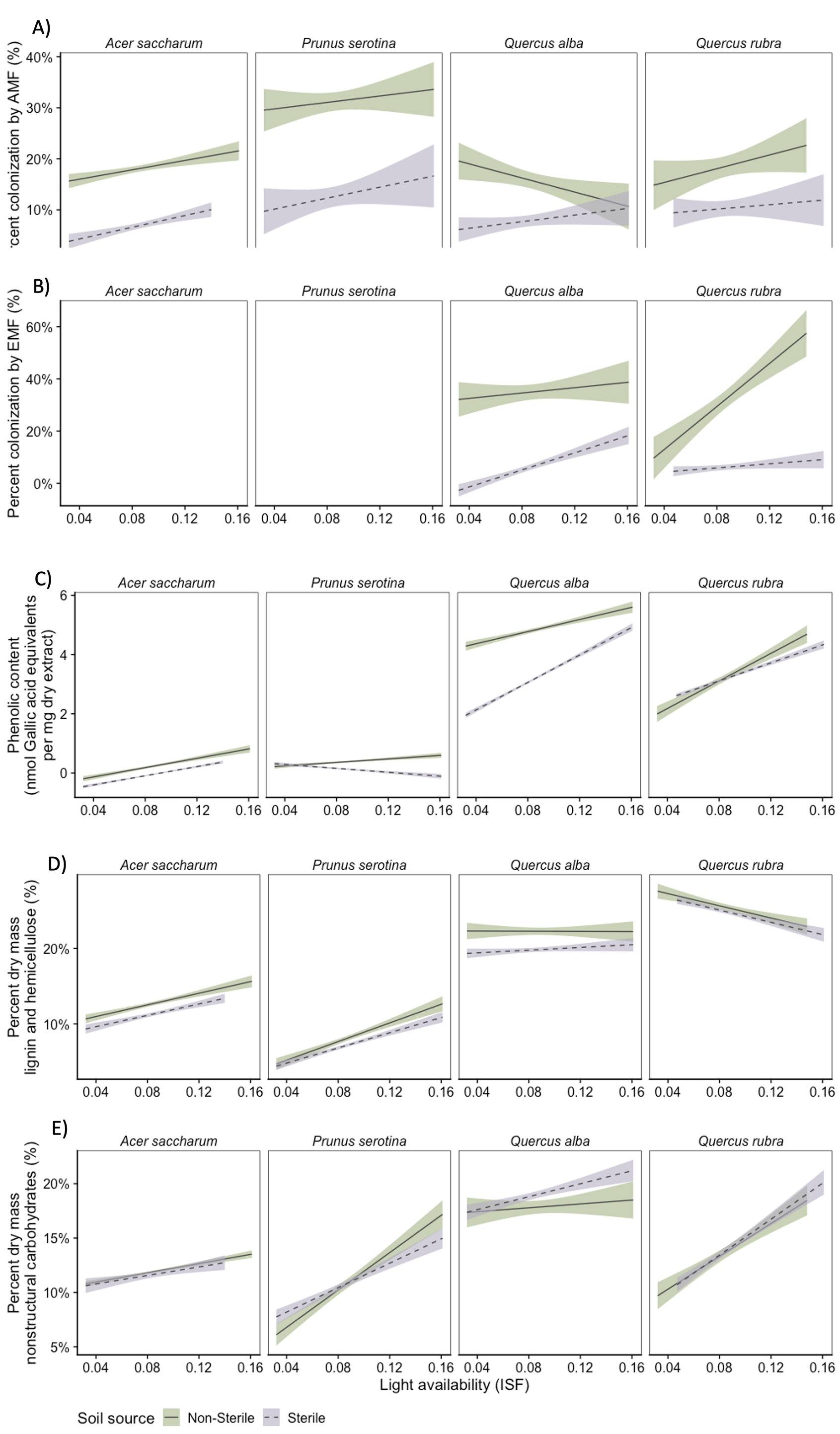

Supplement: S6 Fig — A) AMF colonization, B) EMF colonization, C) phenolics, D) lignin, E) NSC. (TIF) [file pone.0293906.s006.tif]

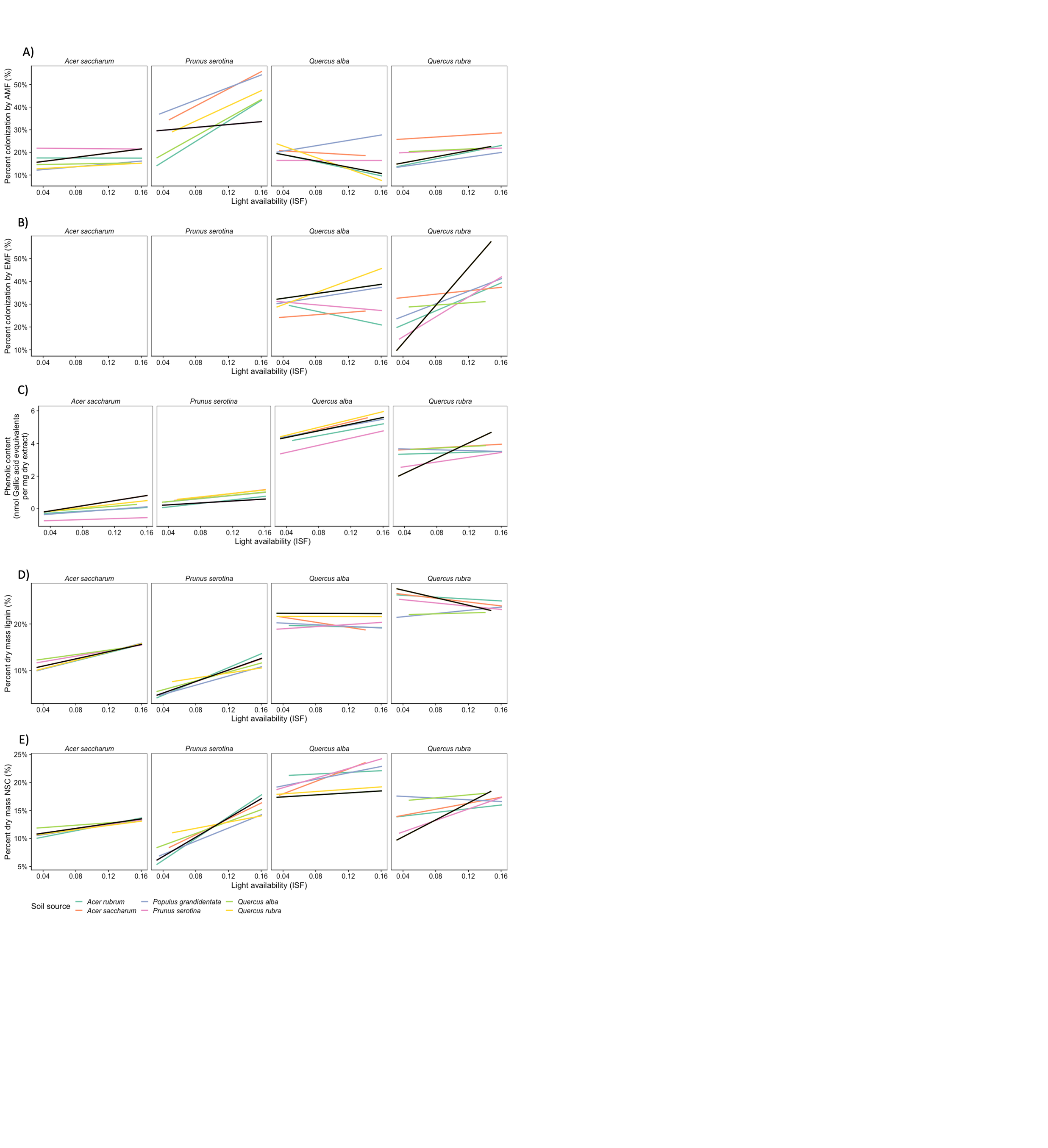

Supplement: S7 Fig — A) AMF colonization, B) EMF colonization, C) phenolics, D) lignin, and E) NSC. (TIF) [file pone.0293906.s007.tif]

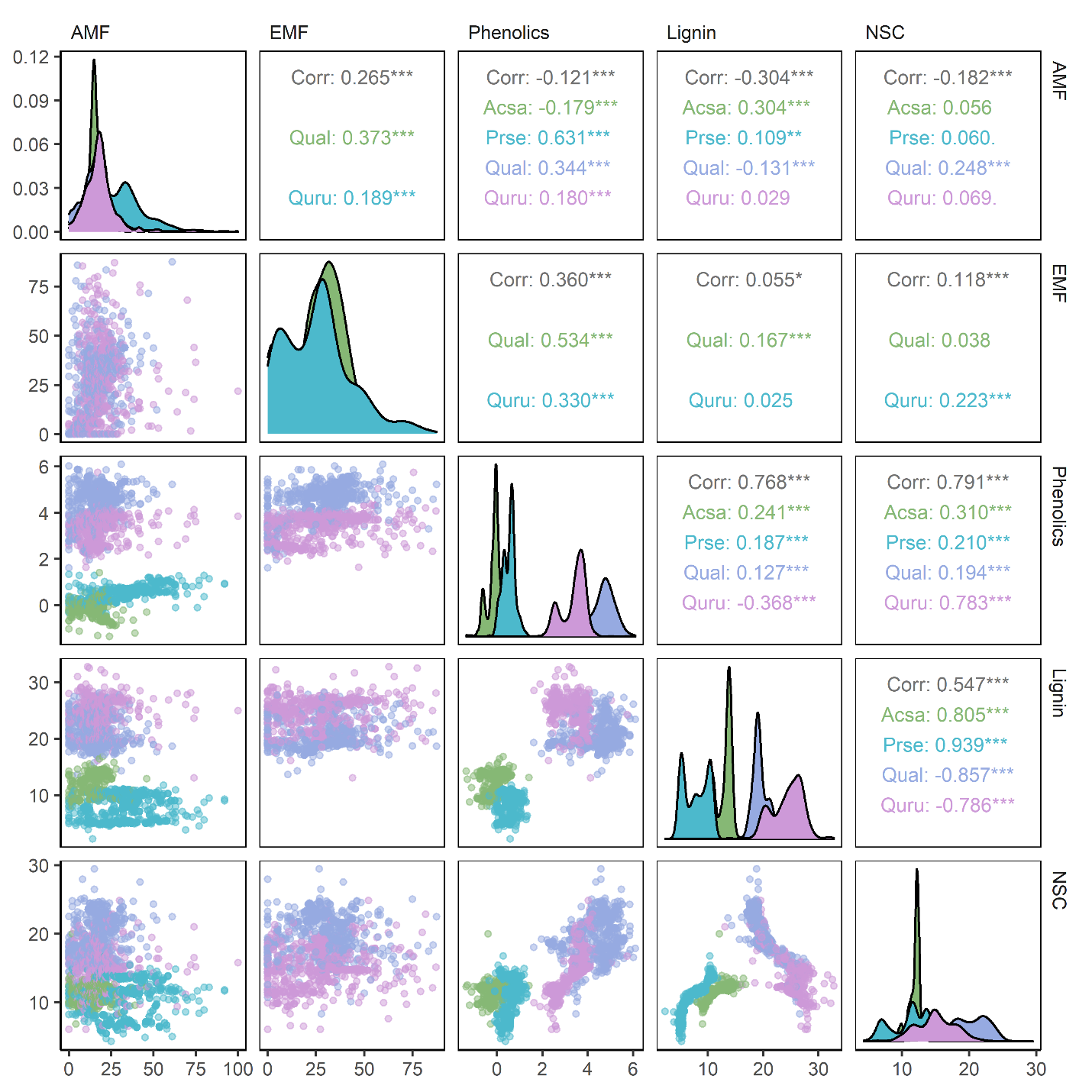

Supplement: S8 Fig — (TIF) [file pone.0293906.s008.tif]

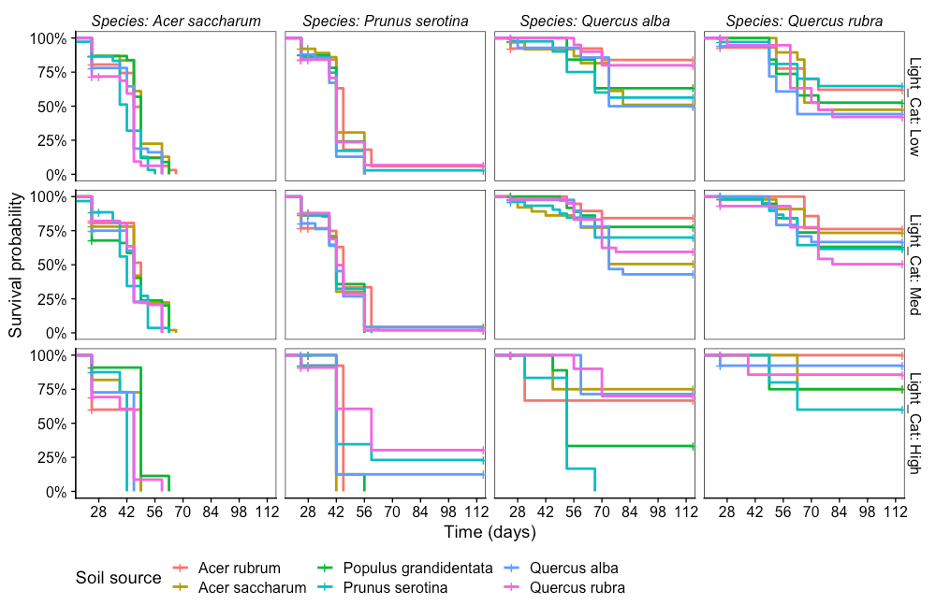

Supplement: S9 Fig — (TIF) [file pone.0293906.s009.tif]

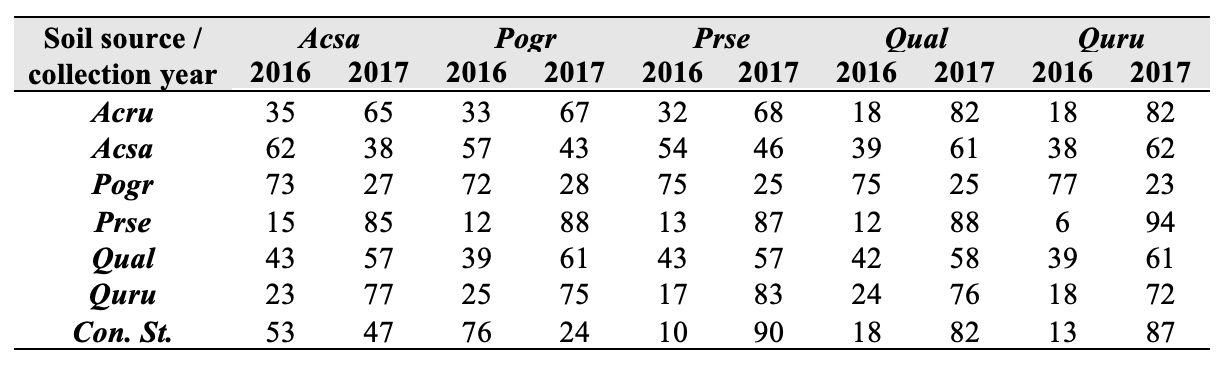

Supplement: S1 Table — For each seedling species and soil source. We collected intact soil cores during the summers of 2016 and 2017. Before transplanting the soil cores into the common garden field plots, we stored the cores inside the research field station. Cores were stored after mesh had been glued to the bottom and 2 open sides of the enclosing pots. We covered the open top of the pot with a plastic lid specifically designed to fit our pots. This ensured that there was no potential for contamination of the pots before moving them back to the field. (TIF) [file pone.0293906.s010.tif]

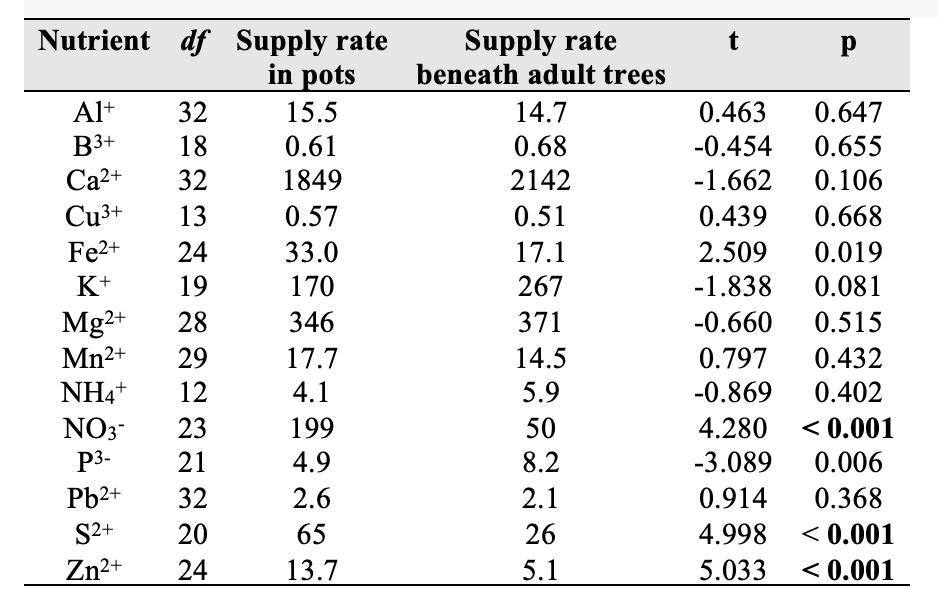

Supplement: S2 Table — Bolded values are significant at p < 0.0036, Bonferroni-corrected to α = 0.0036, for original α = 0.05 and n = 14 tested nutrients. (TIF) [file pone.0293906.s011.tif]

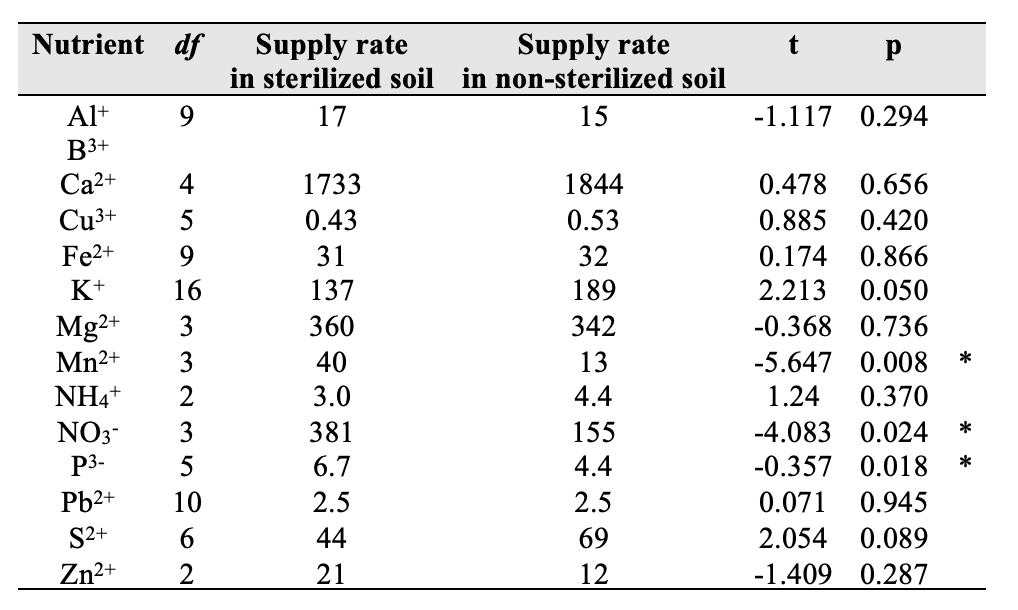

Supplement: S3 Table — Alpha was Bonferroni-corrected to α = 0.0038, for original α = 0.05 and n = 13 tested nutrients. p-values marked with * are marginally significant at original α = 0.05. (TIF) [file pone.0293906.s012.tif]

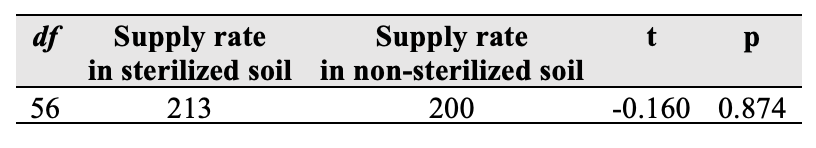

Supplement: S4 Table — For many nutrients, there were not enough replicate pots for a t-test or sample size was small, so we also provide the results of a t-test using the full dataset. (TIF) [file pone.0293906.s013.tif]

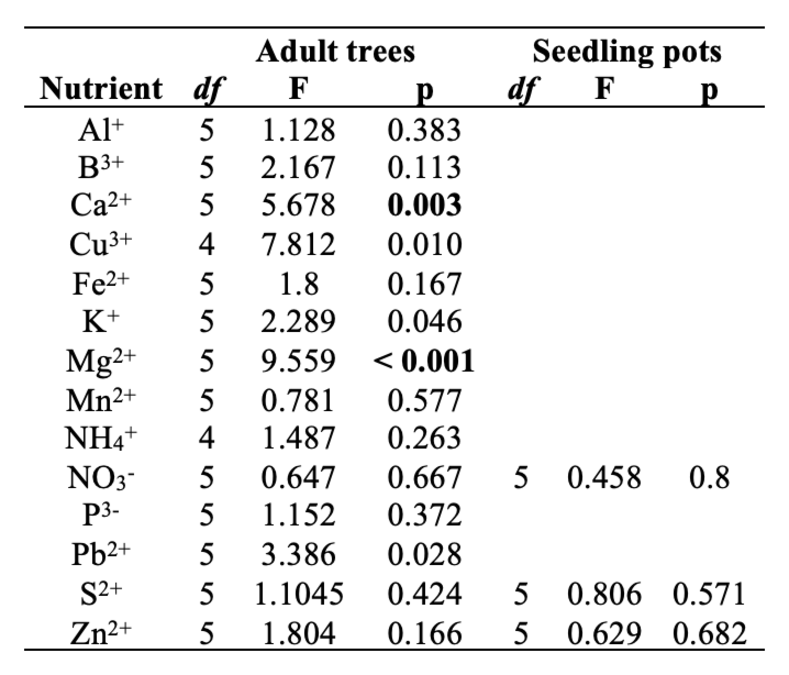

Supplement: S5 Table — Adult trees were used as a proxy for seedling pots, since preliminary analyses showed that, for most nutrients, there were no significant differences between nutrient supply rates for soil in seedling pots versus beneath adult trees. For NO3-, S2+, and Zn2+, there were significant differences in nutrient supply rate for soil in seedling pots versus adult trees, so we also provide results of ANOVAs for seedling pots. Bolded values are significant at α = 0.0036, Bonferroni-corrected for original α = 0.05 and n = 14 tested nutrients. (TIF) [file pone.0293906.s014.tif]

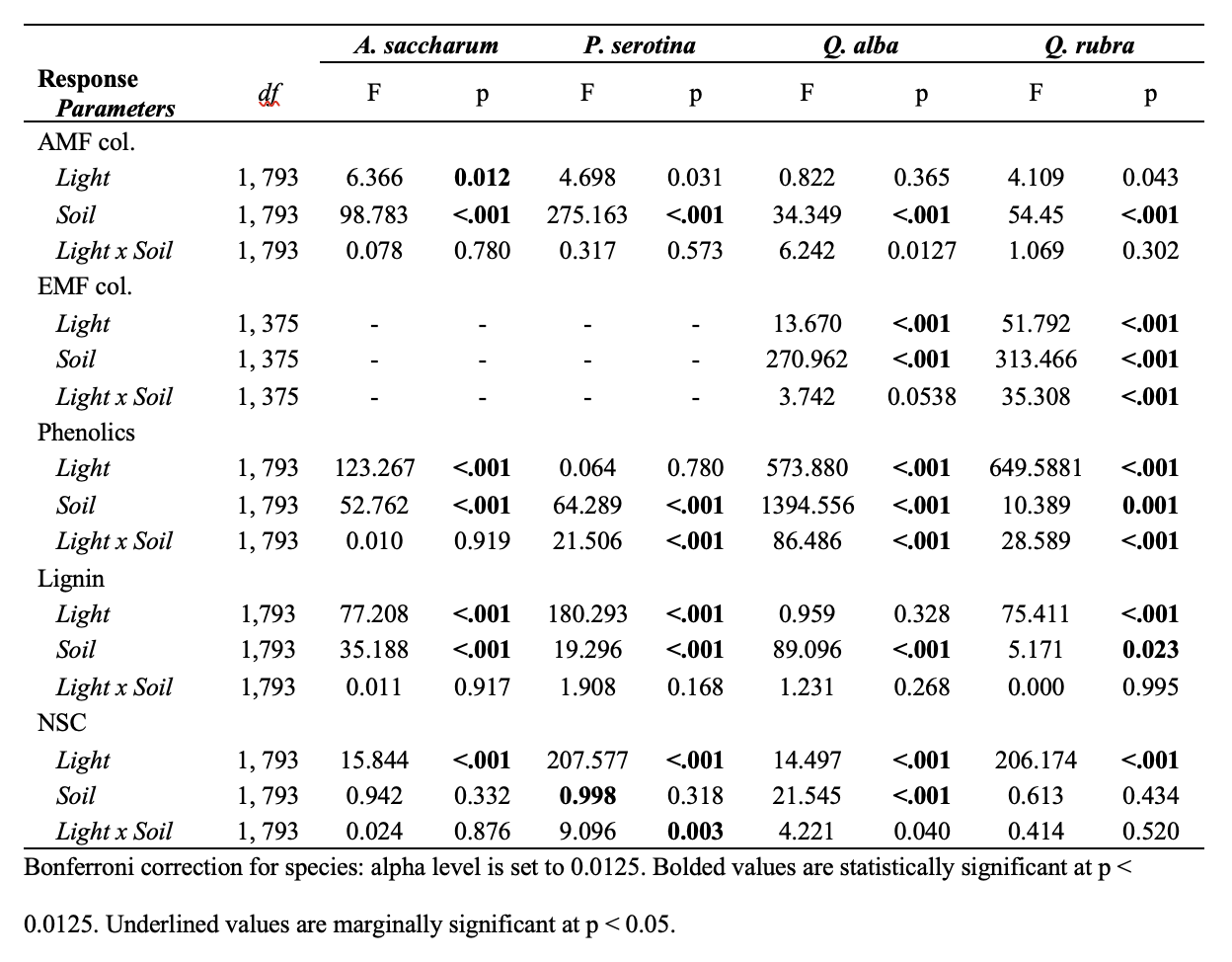

Supplement: S6 Table — AMF colonization, EMF colonization, phenolics, lignin, and NSC. For post-hoc comparisons within species, we used joint tests of estimated marginal means. (TIF) [file pone.0293906.s015.tif]

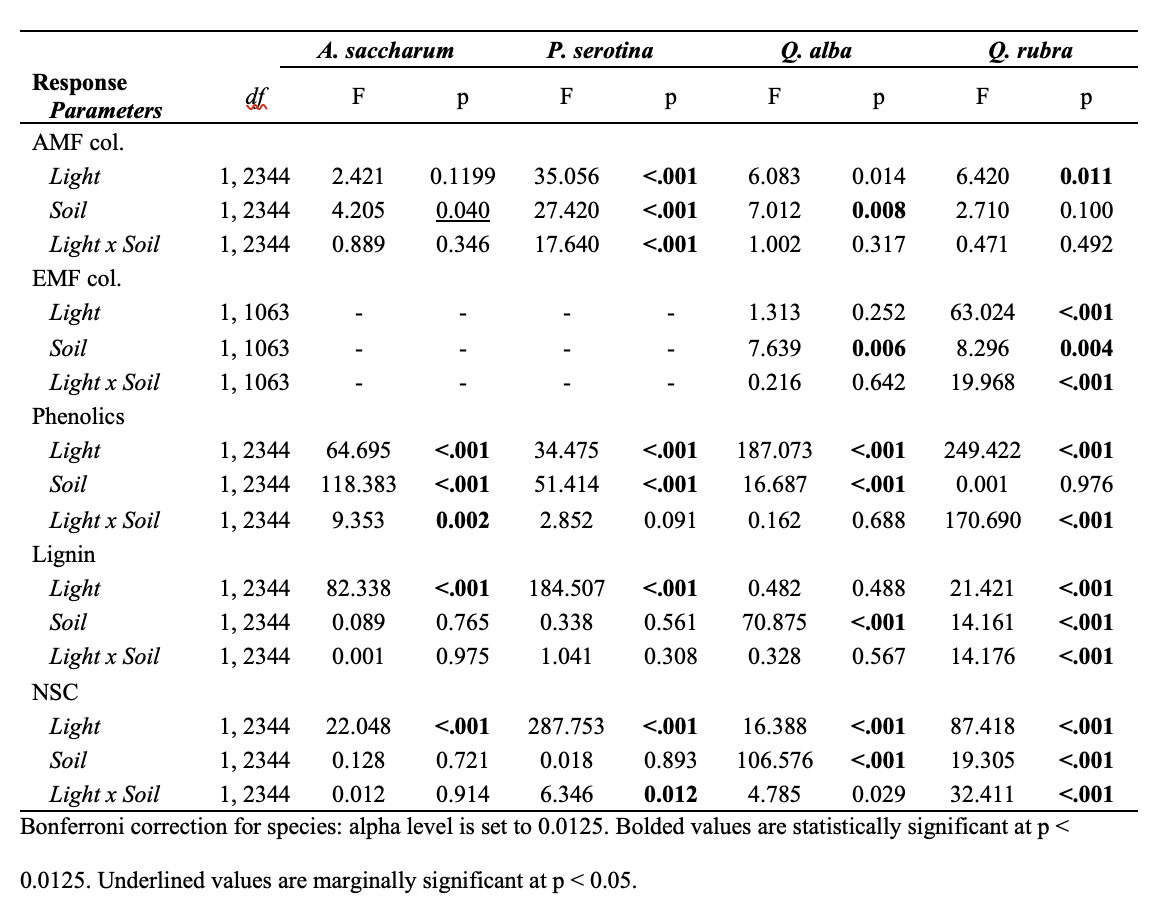

Supplement: S7 Table — Sterilized soil was excluded from the model. For post-hoc comparisons within species, we used joint tests of estimated marginal means. (TIF) [file pone.0293906.s016.tif]

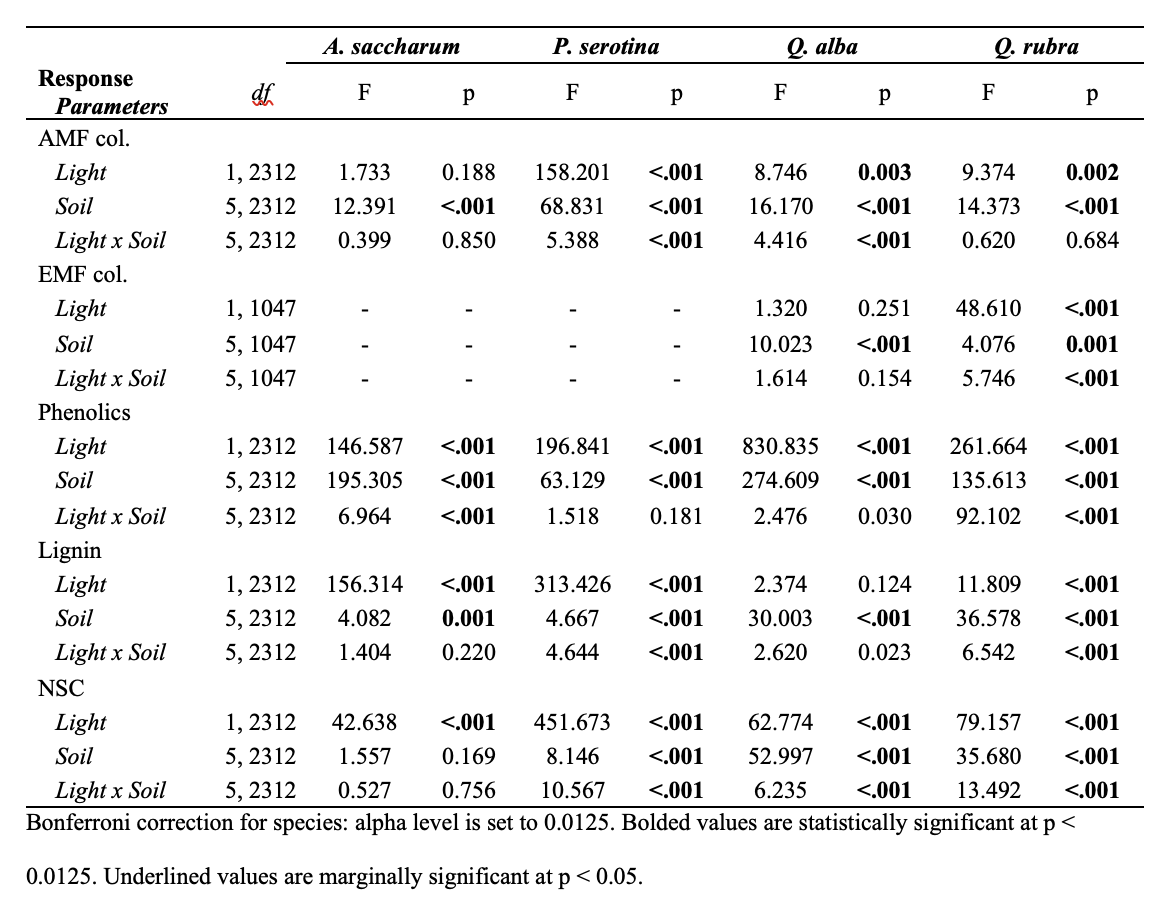

Supplement: S8 Table — Sterilized soil was excluded from the model. For post-hoc comparisons within species, we used joint tests of estimated marginal means. (TIF) [file pone.0293906.s017.tif]

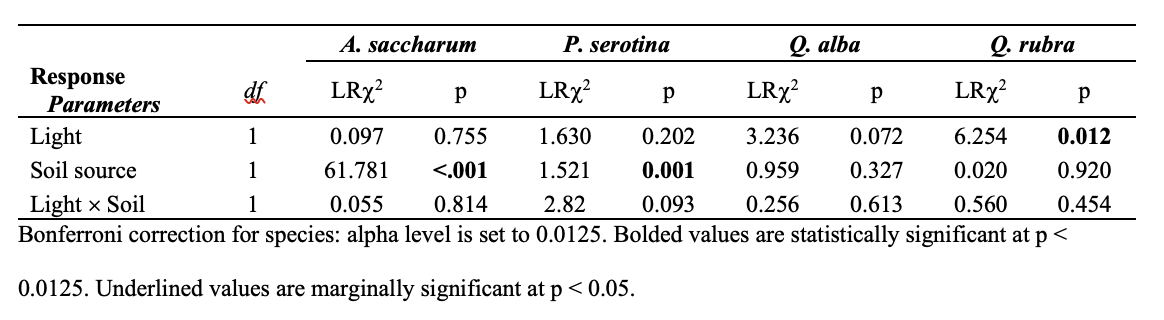

Supplement: S9 Table — Individual models were performed for each species. (TIF) [file pone.0293906.s018.tif]

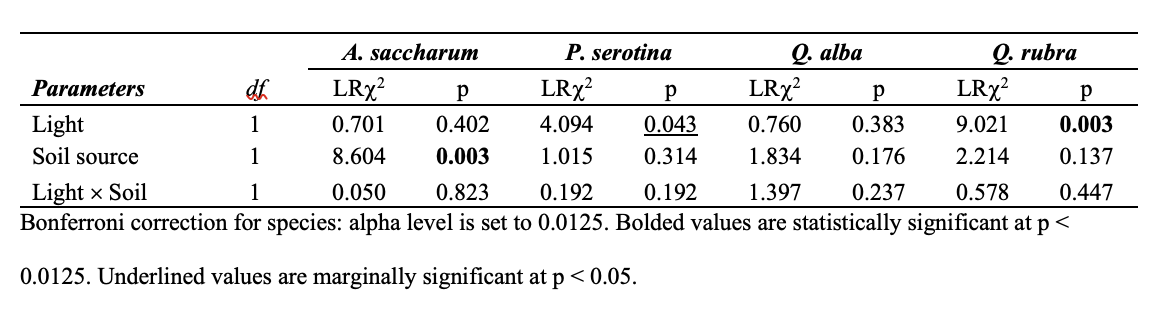

Supplement: S10 Table — Individual models were performed for each species. (TIF) [file pone.0293906.s019.tif]

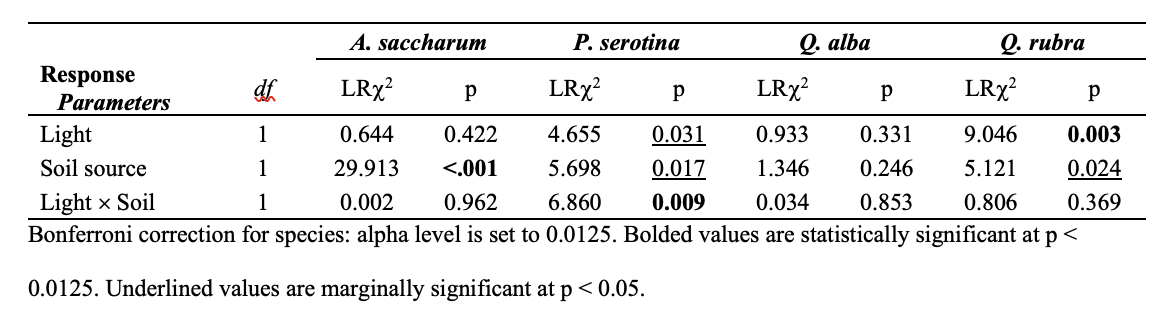

Supplement: S11 Table — Individual models were performed for each species. Sterilized soil was excluded from the model. (TIF) [file pone.0293906.s020.tif]
