# Supplementary material for: Tree seedling functional traits mediate plant-soil feedback survival responses across a gradient of light availability
Source: PLoS One. 2023 Nov 27;18(11):e0293906. doi: 10.1371/journal.pone.0293906 (PMC10681222; doi:10.1371/journal.pone.0293906)
Supplement: S1 File — (DOCX) [file pone.0293906.s021.docx]

**Nutrient availability**

We tested if there were differences in soil nutrient supply rates (e.g., NH_4_^+^, NO_3_^−^, PO_4_^3−^, K^+^, SO_4_^2−^, Ca^2+^, Mg^2+^, Al^3+^, Fe^3+^, Cu^+^, Zn^+^, B^3+^, Mn^2+^, Pb^4+^, and Cd^2+^) by soil source treatments (between undisturbed soil under adult trees where soil was collected and in the fungal exclusion pots) and sterilization treatment (non-sterilized vs. sterilized conspecific soil in fungal exclusion pots) with plant root simulator (PRS™) probes (Western Ag Innovations Inc., Saskatoon, SK) at 0-7 cm depth. Four replicate PRS probes were installed under the adult trees we collected soil from (6 adults trees for *Acer saccharum* and *Quercus rubra* and 3 adult trees for *Acer rubrum*, *Populus granditentata*, *Prunus serotina* and *Querucs alba*). In addition, PRS probes were installed in 240 fungal exclusion pots planted with *A. saccharum* seedlings [(6 non-sterilized soil sources x adult trees (6 for *Acer saccharum* and *Quercus rubra* and 3 for the other 4 tree species) x 2 light treatments (low and high light field plots) x 4 seedling replicates) + (*Acer saccharum* sterilized soil x 6 adult trees x 2 light treatments x 4 seedlings replicates)]. PRS probes were installed 3-wks after planting and harvested 3-wks later.


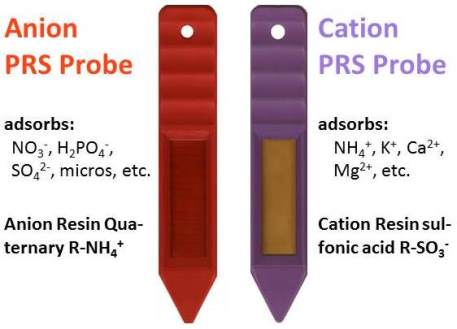
“PRS probes are ion exchange resin membranes held in plastic supports that are easily inserted into soil to measure ion supply *in situ* with minimal disturbance. Anion probes have a positively-charged membrane to simultaneously attract and adsorb all negatively-charged anions... Cation probes have a negatively-charged membrane to simultaneously attract and adsorb all positive-charged cations… Prior to use, ion exchange membranes are saturated with a counter-ion that is easily desorbed, allowing ready absorption of soil ions. Anion probes are saturated with HCO_3_^-^ and cation probes are saturated with Na^+^. When buried, soil ions displace the counter-ions at a rate that depends on their activity and diffusion rate in soil solution. The quantity of soil ions adsorbed during a burial period is a function of all soil properties (physical, chemical, and biological) controlling nutrient availability in soil.” (<https://www.westernag.ca/innovations/technology/basics>)

Due to systemic error in lab processing, sample sizes for some of the treatments were greatly reduced. Subsequent t-tests were conducted with pooled datasets at the nutrient or species level, rather than paired t-tests at the adult tree level.
