# Supplementary material for: Tree seedling functional traits mediate plant-soil feedback survival responses across a gradient of light availability
Source: PLoS One. 2023 Nov 27;18(11):e0293906. doi: 10.1371/journal.pone.0293906 (PMC10681222; doi:10.1371/journal.pone.0293906)
Supplement: S2 File — (DOCX) [file pone.0293906.s022.docx]

**Percent colonization by mycorrhizal fungi**

To quantify mycorrhizal colonization, prior to drying seedlings, 5-10 root fractions per individual (1cm sections of wet root), were retained, weighed, and stained with 5% Schaeffer black in in vinegar solution [1]. Percent root colonization by AMF was quantified by inspecting 100 intersections between the microscope eyepiece crosshairs and roots for AMF structures (i.e., vesicles, arbuscules, coils, and hyphae) every 1mm at 200x magnification [2]. AMF fungal structures were distinguished from other fungi that can inhabit the root interior (e.g., dark septate fungi) by comparing slides to established reference images. Percent root colonization by EMF was quantified by counting the number of intact root tips with and without Hartig nets at 100x magnification every 2mm along the root until 100 root tips were scored.

**Phenolics**

To quantify phenolics, we collected hypocotyl samples, cut into <1mm pieces. We extracted phenolics in 5mL methanol in the dark for 16 hours at room temperature. The methanol extracts were filtered and adjusted to 5mL, and then we quantified total phenolics using a microplate-adapted colorimetric total phenolics assay with Folin-Ciocalteu reagent [3,4].

**Lignin**

To quantify lignin, root and stem samples were lyophilized and coarsely ground at 1mm using a Wiley Mill. We ran 0.5g root and stem samples through a series of extractions using an ANKOM Fiber Analyzer (ANKOM Technologies, Macedon, NY, USA). We used a Neutral Detergent Fiber extraction to wash off soluble cell contents (e.g., carbohydrates, lipids, pectin, starch, and soluble proteins). We then used an Acid Detergent Fiber extraction with 1.00 normal sulfuric acid to wash off hemicellulose and bound proteins and an Acid Detergent Lignin extraction with 72% sulfuric acid to wash off cellulose, leaving only lignin and recalcitrant materials. Finally, we ashed the samples to quantify dry mass lignin.

**Nonstructural carbohydrates**

To quantify nonstructural carbohydrates (NSC), we analyzed stem samples, using a standardized enzyme method for sugar and starch extraction and quantification [5,6]. We dried seedling stems and peach leaf standard reference material (MillporeSigma-NIST1547) at 60°C overnight to remove moisture. We then weighed out 30mg of each for analysis and separated sugars and starches with hot ethanol extraction. We used α-amylase and amyloglucosidase to convert starch to glucose. We quantified sugars using phenol-sulfuric acid colorimetric assay and starches using a glucose-hexokinase colorimetric assay (MillporeSigma-GAK20). We calculated total NSC concentrations as the sum of soluble sugar and starch concentrations derived from the assays.

**References**

1. Vierheilig H, Coughlan AP, Wyss U, Piché Y. Ink and vinegar, a simple staining technique for arbuscular-mycorrhizal fungi. Appl Environ Microbiol. 1998;64: 5004–5007. doi:10.1128/AEM.64.12.5004-5007.1998

2. McGonigle TP, Miller MH, Evans DG, Fairchild GL, Swan JA. A new method which gives an objective measure of colonization of roots by vesicular—arbuscular mycorrhizal fungi. New Phytologist. 1990;115: 495–501. doi:10.1111/j.1469-8137.1990.tb00476.x

3. Ainsworth EA, Gillespie KM. Estimation of total phenolic content and other oxidation substrates in plant tissues using Folin–Ciocalteu reagent. Nat Protoc. 2007;2: 875–877. doi:10.1038/nprot.2007.102

4. Waterman P, Mole S. Analysis of phenolic plant metabolites. Blackwell Scientific; 1994.

5. Quentin AG, Pinkard EA, Ryan MG, Tissue DT, Baggett LS, Adams HD, et al. Non-structural carbohydrates in woody plants compared among laboratories. Tree Physiology. 2015;35: 1146–1165. doi:10.1093/treephys/tpv073

6. Landhäusser SM, Chow PS, Turin Dickman L, Furze ME, Kuhlman I, Schmid S, et al. Standardized protocols and procedures can precisely and accurately quantify non-structural carbohydrates. Tree Physiology. 2018;38: 1764–1778. doi:10.1093/treephys/tpy118
