# Supplementary material for: Tree seedling functional traits mediate plant-soil feedback survival responses across a gradient of light availability
Source: PLoS One. 2023 Nov 27;18(11):e0293906. doi: 10.1371/journal.pone.0293906 (PMC10681222; doi:10.1371/journal.pone.0293906)
Supplement: Attachment — Submitted filename: Response to Reviewers 2.docx [file pone.0293906.s024.docx]

**Response to reviewers**

To whom it may concern,

Please find attached a second revised version of the manuscript titled “Tree seedling functional traits mediate plant-soil feedback survival responses across a gradient of light availability”. We believe that the manuscript has been strengthened through the revisions we made, based on the suggestions from editors and external reviewers. We include below the reviewers’ comments and a summary of how we revised the manuscript to address these concerns. Please feel free to contact us if you have questions or need clarification on the revised version.

Thank you for your consideration. We look forward to hearing your opinion of our revised manuscript.

Sincerely,

Katherine E. A. Wood

Ph.D. Candidate

Department of Forestry

Graduate Program in Ecology, Evolutionary Biology, and Behavior

Michigan State University

woodkat7@msu.edu

**Reviewer #1:** All of my concerns were adequately addressed.

**Reviewer #2:** Although the authors have addressed one of my two initial concerns about the manuscript by updating their figures (and including a conceptual diagram: that is very helpful!), my concern regarding the presentation of overall survival results remains. In their response, the authors indicated that on the new versions of the figures there would be an indication of the group level survival; however, in the version I received there is still no reporting or discussion of the group level, overall survival. This is a crucial result that cannot be omitted; it is necessary for the interpretation of all other results.

The authors include language like "although the majority of A. saccharum and P. serotina died by the end of the growing season" (line 486), however, it seems from the figures that 100% of these seedlings died. I encourage the authors to simply include a table that states the overall survival at the end of the season of each species in each group (soil source and light), and to directly refer to this in the results and discussion. For example, if none of the A. saccharum seedlings survived, this must be noted, as it weakens (but doesn't completely discount) the assertion that this species exhibited PSF.

We thank the reviewer for their feedback. We have added a new table (Table 2, Line 288) that states the overall survival (number of seedlings and percentage) at the end of the season for each species in each group (soil source and light). Because there was not a significant interaction effect between soil source and light availability on seedling survival, we presented the results for soil source and light availability separately. Table section A aligns with figure 2A, and table section B aligns with figure 2B. In addition, we added a paragraph about these results to the discussion (Line 398).
